# Supplementary material for: The citation advantage of linking publications to research data
Source: PLoS One. 2020 Apr 22;15(4):e0230416. doi: 10.1371/journal.pone.0230416 (PMC7176083; doi:10.1371/journal.pone.0230416)
Supplement: Attachment — Submitted filename: Reviews.docx [file pone.0230416.s002.docx]

Journal Requirements:

2. We note that Iain Hrynaszkiewicz is affiliated with the Public Library of Science (PLOS). To ensure transparency, please include this information in the Competing Interests statement.

Done.

3. Thank you for stating the following in the Financial Disclosure section:

This work was supported by The Alan Turing Institute under the EPSRC grant EP/N510129/1 and by Macmillan Education Ltd, part of Springer Nature, through grant RG92108 "Effect of data sharing policies on articles' citation counts" both granted to BMC and IH.

Done.

We note that one or more of the authors are employed by a commercial company: Macmillan Education Ltd

1. Please provide an amended Funding Statement declaring this commercial affiliation, as well as a statement regarding the Role of Funders in your study. If the funding organization did not play a role in the study design, data collection and analysis, decision to publish, or preparation of the manuscript and only provided financial support in the form of authors' salaries and/or research materials, please review your statements relating to the author contributions, and ensure you have specifically and accurately indicated the role(s) that these authors had in your study. You can update author roles in the Author Contributions section of the online submission form.

Please also include the following statement within your amended Funding Statement.

“The funder provided support in the form of salaries for authors [insert relevant initials], but did not have any additional role in the study design, data collection and analysis, decision to publish, or preparation of the manuscript. The specific roles of these authors are articulated in the ‘author contributions’ section.”

If your commercial affiliation did play a role in your study, please state and explain this role within your updated Funding Statement.

Done.

2. Please also provide an updated Competing Interests Statement declaring this commercial affiliation along with any other relevant declarations relating to employment, consultancy, patents, products in development, or marketed products, etc.

Within your Competing Interests Statement, please confirm that this commercial affiliation does not alter your adherence to all PLOS ONE policies on sharing data and materials by including the following statement: "This does not alter our adherence to  PLOS ONE policies on sharing data and materials.” (as detailed online in our guide for authors [http://journals.plos.org/plosone/s/competing-interests](https://eur01.safelinks.protection.outlook.com/?url=http%3A%2F%2Fjournals.plos.org%2Fplosone%2Fs%2Fcompeting-interests&data=02%7C01%7Cgcolavizza%40turing.ac.uk%7Cf3f7cec5ffde4c09503608d72fa31985%7C4395f4a7e4554f958a9f1fbaef6384f9%7C0%7C1%7C637030250712452772&sdata=ZF2E4S2GLtm4Yt023YN7TxzE5Ov%2FR%2FOix%2FwXvPF27wE%3D&reserved=0)). If this adherence statement is not accurate and  there are restrictions on sharing of data and/or materials, please state these. Please note that we cannot proceed with consideration of your article until this information has been declared.

Please include both an updated Funding Statement and Competing Interests Statement in your cover letter. We will change the online submission form on your behalf.

Please know it is PLOS ONE policy for corresponding authors to declare, on behalf of all authors, all potential competing interests for the purposes of transparency. PLOS defines a competing interest as anything that interferes with, or could reasonably be perceived as interfering with, the full and objective presentation, peer review, editorial decision-making, or publication of research or non-research articles submitted to one of the journals. Competing interests can be financial or non-financial, professional, or personal. Competing interests can arise in relationship to an organization or another person. Please follow this link to our website for more details on competing interests: <http://journals.plos.org/plosone/s/competing-interests>

Done/verified.

5. Review Comments to the Author

**Editor:**

MINOR ISSUES

The first reviewer asks for clarifications and details to be added to different sections of your manuscript. This reviewer also rightly notes that the use of causal terms is not warranted given the correlational data that you used. The second reviewer also provided useful feedback (als in his attached PDF) that could help you improve the writing and details of the manuscript. Finally, I wondered myself whether you could add more details on the citation data in relation to the well known Scopus and ISI Web of Science databases.

Several of the suggested edits have been integrated.

Regarding WoS or Scopus citation data, an assumption of our study is that citation counts and derived statistics (such as H-indexes) calculated from the PubMed OA dataset are correlated to global citation counts as captured by WoS or Scopus. We agree this is critical, and hence we proceeded to test this assumption for PLOS ONE publications using Web of Science. We were able to match 199,304 publications over 203,307 using their DOI. We compared the WoS global citation counts (updated to Summer 2019) with the global citation counts from our dataset (updated to December 2018 included). We find a positive correlation of 0.83 and that citation counts from PubMed OA on average account for 18% of the global ones from WoS. A scatter plot with citation counts from PubMed OA on the y axis (n_cit_tot) and from WoS on the x axis (n_cits) is reported below. We added a footnote in the updated manuscript to report this check, as we believe it supports our assumption as stated above. Unfortunately, we cannot add these data to the repository.

MAJOR ISSUE

The second reviewer was rather critical of your analyses. I agree with this reviewer that many different models could potentially be run and that the choice of analysis can affect the key result of the citation advantage. It is important that you do not oversell the results (not necessary here at PLOS) and that you avoid the issue of overfitting (or any selection of models that could inflate the citation advantage). Moreover, I agree with this reviewer that given the typically very skewed distribution of citation data, it would be better to use negative binomial regressions (or a zero-inflated Poisson model) instead of normal regressed based on the log of number of citations (plus 1). I ask you to consider carefully the statistical issues raised by this reviewer and re-run the key models to ensure that the outcomes are robust against the use of alternative statistical and predictive models.

We believe that quantifying the average increase in citations a paper with a DAS has in the abstract is quite important to convey the whole point of the analysis. Extensive checks have been carried out to verify the soundness of these results. More details below.

We thank both reviewers for their remarks and suggestions.

**Reviewer #1:**

This article describes a rigorous and technically sound research effort to examine the content of a large sample of data availability statements (DAS) reported in PLOS and BMC journals and their association with citation impact. It is highly commendable that the article is accompanied by a detailed repository containing the research data and analysis scripts which should enable verification and re-use (note that other than a cursory examination, I have not actually tried to use these files myself). Limitations of the research design are discussed forthrightly in the discussion section and generally the conclusions seem well calibrated to the research design and the evidence obtained. I only have minor suggestions to improve the clarity of reporting.

“DAS in PLOS” – should the specific PLOS journal be specified?

We consider all PLOS journals in that case, and have clarified in the text.

I suggest that the abstract and introduction mention briefly describe the use of a classifier was used as this was a key part of the method.

Thank you for the suggestion. We have mentioned the classification task in both abstract and introduction, and have stressed its role in the study.

“We established a set of rules to detect and extract statements from both sets of journals, as documented in our repository” – it is not obvious where to find the relevant details in the repository and more details in manuscript would be helpful. The repository is commendably detailed, but linking directly to the relevant section will help readers find the correct information more quickly.

This is right, yet unfortunately this kind of information is often considered as a technicality by readers, and the article is already quite detailed. The relevant code is to be found here (lines 228-246): <https://github.com/alan-turing-institute/das-public/blob/master/dataset/parser_function.py>.

“We identified four categories of DAS, further described in Table 1. We use fewer categories than [16], mainly due to the sparsity of most of them” – the referenced study is based on PLOS, is there good reason to believe these statement types are also sparse in the BMC dataset?

Indeed, we tested a finer-grained classification first, on both BMC and PLOS articles, but the classifiers could not catch the differences between some of them, especially those with few examples. We thus grouped them.

Description of classifier results – Table 2 and corresponding text – I think further explanation would be very helpful here. I don’t think the headings in Table 2 are clearly defined and the text refers to ‘accuracy’ which doesn’t appear in the table. As far as I can tell, the results pertain to sensitivity, but not specificity i.e., do we know how many DAS the classifier might miss? Also, if I have understood correctly, the training set and test set were specifically populated with DAS from four pre-selected categories. If so, how do we know how the classifier will perform in the wild? (i.e., when handling other DAS types).

We have reported overall accuracy of the classified in the text, while Table 2 reports a break-down of precision, recall (=sensitivity), and F1 score by category. Specificity per class is now included. We have assumed that the four categories are the only DAS types worth analysing for our purposes (also see above).

Figure 2 – I suggest the legend includes actual category descriptions/names rather than “class 1” etc. Otherwise the reader has to keep moving between the main text and the figure.

Done.

Generally, the conclusions are well calibrated to the evidence. However, occasionally, language that implies a causal relationship is used when that is not warranted by the present research design e.g., “all DAS categories positively impact citation counts”

We have rephrased.

**Reviewer #2:**

This is a review of Colavizza et al “The citation advantage of linking publications to research data”, as submitted to PLOS ONE. The article addresses the hypothesis that the type of Data Availability Statement (DAS) included at the end of a published paper affects its subsequent citation rate.

The authors gather a very large dataset of Open Access articles from the PubMed OA collection, all from either PLOS or BMC journals. They use Natural Language Processing to extract the DAS and then classify them into four categories according to their level of detail. The authors then apply a linear model that includes many other variables known to influence citation count (e.g. time since publication, author popularity, and manuscript field). After accounting for these other variables they find a small but significant boost from including a DAS, particularly the most detailed category (3).

This is a decent article and will become a useful contribution, but I would like the authors to address the following points.

First, the model fitting is the weakest aspect of the article. The red flag for me is in the abstract, “can have up to 25.36% higher citation impact”, as this phrasing sounds like the authors tried many different models before arriving at one that shows the largest effect of DAS on citations. This might also explain why the model presented in the article contains both h_index_mean and h_index_median, but leaves out journal. I appreciate that decisions about model building are difficult, and reviewers always want some variable included/excluded, but the authors need to avoid the appearance of p-hacking; this is best done by deciding exactly what question(s) the article is testing and focus on one or two models that test it most efficiently (even if the outcome is less compelling).

We are rather surprised by this comment of the reviewer. We have made several efforts to motivate the choice of variables in the model and to test the robustness of our results using different modelling approaches (footnote 6: “The models we test include ANOVA, tobit and GLM with negative binomial (on the full dataset and on the dataset of papers with 1 or more received citations), zero-inflated negative binomial, lognormal and Pareto 2 family distributions. We further test different models, including logistic regression on DAS category and on whether or not a paper is cited at least once.”). Given comparable outcomes, and not slightly higher p-values (which are always significant to numerical precision), we selected the model that is the easiest to interpret, and which is suggested by the relevant literature (references 60 and 61), and included a robust version to further verify overfitting. Furthermore, we checked the following: 1) journal, noting that the use of subject areas accounts for part of that signal, 2) ablation checks (e.g., mean and media H-index), 3) interactions. Everything is documented and/or can easily be reproduced from our repository, as we agree that the paper should focus on one or two models and leave these further details out.

A more robust approach that is used in the medical literature for similar circumstances (i.e. observational data) is as follows. First, estimate the strength of the relationship between your central variable of interest (DAS category) and citations. This will approximately estimate the amount of variance in citations that DAS category could explain. Next, present the full model, including all the covariates that could also affect citation count, either independently (e.g. year) or through their effects on DAS (e.g. H-index, where more meticulous researchers have both better DAS and better articles, and hence more citations). The effect size associated with DAS category will certainly go down, but what matters here is by how much: a large drop implies that the other covariates were driving most of the initial effect size, whereas a small decrease shows that DAS category itself has a robust effect on citations.

We appreciate the suggestion. Tha same model (1) just with DAS categories shows category 3 with a coefficient of 0.296, which goes to 0.252 in the full model, hence corroborating a robust effect. Coefficients are lower and their drop proportionally larger for categories 1 and 2. We added these results in the paper.

The authors should be also using a mixed effects model, where articles are nested within journal, allowing journal identity to act as a random effect. This approach also eliminates the need for a separate subject area and publisher variables. The extremely wide scope of PLOS ONE presents some difficulties, but the authors could consider splitting PLOS ONE articles up by their broad subject category (e.g. PLOS_ONE_biology_and_life_sciences), and using this as journal identity for these articles instead.

Thank you for the suggestion. We added more code to the repository testing a mixed effects model with articles nested within journal, as suggested. We tested two models, one assuming normally distributed data and using the transformed citation counts, as in the main reported model, and another assuming lognormally distributed data (we motivate these two choices by further fitness checks over a variety of possible distributions, which we also include in the repository). We tested both models twice, on data filtering out articles and journals with less than 100 and 500 articles to avoid sparsity effects. All models consistently point to a significant positive effect of DAS category 3 on citation counts. We added these further tests to footnote 6.

Since citations are count data and the mean is close to zero (Table 4), the model should be using a Poisson distribution and not a normal distribution. Using a normal model under these circumstances may lead to odd results, such as predicting negative citations  (which are clearly impossible). If the majority of articles have zero citations, it may be better to use a negative binomial model or even a zero-inflated Poisson model. Moreover, x-axis variables should remain untransformed unless there is a specific a priori reason to transform them, or if transforming them corrects a serious issue with the distribution of the residuals: one cannot put in both the transformed and the untransformed covariates and pick the one that looks best by p-value. Note that it’s the model residuals that need to be normally distributed, and not the covariates themselves.

Please see above. The repository contains code to check model residuals. We also refer the reviewer to references 60 and 61, where the problem of modelling citation counts and their typical distribution with a high fraction of zeros is discussed.

The authors may also want to consider including subsequent analyses that home in on particular subsets of the data. For example, the types of DAS encountered seems to differ quite significantly between the ‘encouraged’ and the ‘mandatory’ time period, with high quality category 3’s dominating the former. These category 3 DAS likely come from competent, highly motivated groups, and these are expected to get more proportionally more citations even in the absence of the DAS. Analysing the ‘encouraged’ and ‘mandatory’ data separately would help readers better understand this difference. As it is, I was left with the suspicion that the significant citation advantage for the category 3 DAS in the main model is driven principally by the ‘encourage’ part of the dataset.

The fact that an article is published under an encouraged or mandated policy is already captured by boolean variables in the models.

I was also (initially) puzzled why the authors include year and month of publication as separate variables. They state that there is a “known advantage of publications published early in the year” (line 243), but don’t provide a reference. Is this advantage above and beyond that expected because an article published in January is 11 months older than one published in December of the same year? If this advantage really does exist, does the reader really need to worry about it? My initial idea was to recommend having ‘months since publication’ as a single variable, as it’s simpler and easier to understand, but it seems that the citation data are calculated only for articles that appear 36 months after publication of the focal article, so ‘months since publication’ is constant. It would be worth making this a bit clearer in the article. Minor point: does the number of citations in the dataset grow each year just because the number of OA articles published each year is growing? Or is the number of references in each article increasing too?

This is correct. The month issue is indeed a minor one: on the one hand, as the reviewer notes, the advantage might be given by the binning effect of the year. On the other hand, several articles lack the month information and are thus assigned the value 6 as default (June). It is indeed the case that both the number of citations and the length of reference lists is rising over time. The rise of citations might be happening proportionally more quickly, given that the number of articles is also increasing, but we lack the full counts for them obviously.

I’ve made a number of other suggestions directly onto the pdf.

Thank you, we have integrated several in the revised version.
