# Supplementary material for: The citation advantage of linking publications to research data
Source: PLoS One. 2020 Apr 22;15(4):e0230416. doi: 10.1371/journal.pone.0230416 (PMC7176083; doi:10.1371/journal.pone.0230416)
Supplement: Attachment — Submitted filename: response_round_2.docx [file pone.0230416.s003.docx]

Dear Editor,

we attach here the email conversation which followed our second round of reviews, for reference.

To reply to your last message (also below), we added confidence intervals in the abstract, and also revised it to provide for a fuller account of our findings. We also eliminated all footnotes and integrated them into the text or as references, as requested. The text has been edited accordingly where necessary. We believe the modelling part now accounts for the many tests we did, and also reports the limitations of our work.

We finally note that we do not use the best model to provide for results, as discussed in the text. Furthermore, PLoS ONE and other journals have a practice to convey the main findings in the abstract as we do. As a very related example, we can use this recent paper: <https://journals.plos.org/plosone/article?id=10.1371/journal.pone.0225883>. We do this not in an attempt to oversell, but to convey the main finding as effectively as we can.

We hope this is acceptable and that we can get a rapid response.

Thank you,

The authors

Dear Giovanni,

Please accept my apologies for the slow response to your email.

The main issue here concerns both the % statement made in the in the abstract (arguably overly strong) and the lack of pre-registration combined with analytic flexibility that together imply that there is more uncertainty associated with your stated estimate than the abstract and discussion reflect.  Please revise the stated result in the abstract to reflect the uncertainty around this estimate (add a range perhaps?) and add a discussion of the uncertainty of results caused by the option to analyse the current data in many different ways. Much of the latter is already shortly stated in your footnote 6 and detailed in the supplement, but PLOS ONE does not allow footnotes and hence this information should be incorporated and discussed in the main text and detailed in the supplementary file or in an appendix. As stated in my letter: it is better to be safe than sorry, and safety here bears on not overstating the conclusions on what are impressive data and important results. You do not need to “sell” the results here at PLOS; they just need to be right.

I am looking forward to receiving your revision and promise to prioritise handling it amidst my piling to-do-list.

Best wishes,

Jelte Wicherts - Academic Editor PLOS ONE

On 16 Jan 2020, at 13:48, plosone <[plosone@plos.org](mailto:plosone@plos.org)> wrote:

PONE-D-19-18900R1
The citation advantage of linking publications to research data
PLOS ONE

Dear Dr Wicherts,

I'm sorry to bother you again; we realise that you must be busy but the authors of this manuscript are keen to get your advice, following your decision.

We will pass on your response to the authors. If you reply direct to the author, please copy any reply to [plosone@plos.org](mailto:plosone@plos.org)

Kind regards,

Sarah Mills
Staff EO
PLOS ONE

Case Number: 06496119
ref:_00DU0Ifis._5004P129EHJ:ref


--------------- Original Message ---------------

**From:** Giovanni Colavizza [[gcolavizza@turing.ac.uk](mailto:gcolavizza@turing.ac.uk)]
**Sent:** 12/19/2019 7:03 AM
**To:** [plosone@plos.org](mailto:plosone@plos.org)
**Subject:** Re: PLOS ONE Decision: Revision required [PONE-D-19-18900R1] - [EMID:497f802704611836]

Dear Jelte,

Thank you for your message. Before we decide to go into revision, we think it important to clarify what is expected from us. This is particularly crucial as the paper has been considerably delayed already, and it seems some comments are coming from misunderstandings or positions of principle rather than the publication criteria of the journal, and thus we might not be able to address them fully. Please, could you consider my replies to reviewer 2's remarks below and let me know what you think and if you would consider what I propose acceptable for a further revision? Thank you.

Best,

Giovanni

Reviewer #2: Review of PONE-D-19-18900R1 “The citation advantage of linking publications to research data”

This article has been revised in response to the first round of peer review. I’m largely supportive of publication at this point, but I still (strongly) object to the authors’ use of the phrase ‘can have up to’ in the abstract – by focusing their most sensational result they are not accurately reporting what they found.

They should follow their own suggestion in the response to reviewers: “We believe that quantifying the average increase in citations a paper with a DAS has in the abstract is quite important to convey the whole point of the analysis” and focus on the average increase in citations that they found, not the largest increase. Expressing it as a percentage also obscures the relatively small effect size (approx. 1.51 citations after three years with a type 3 compared to 1.14 with type 0)

We already report the model's average (i.e., fitted coefficient effect). The words "can have up to" are just a cautionary way to express it. Maybe the reviewer is suggesting to report an average over all possible models we tested? This would be confusing and, besides, can be reproduced using the available code. Furthermore, we report using the simplest and easiest to understand model we tested, not the one with larger effects, as we remark in the text and we stated in the first rebuttal phase. We also note that reporting effects in this way is totally common. See e.g., [https://elifesciences.org/articles/52646](https://eur01.safelinks.protection.outlook.com/?url=https%3A%2F%2Felifesciences.org%2Farticles%2F52646&data=02%7C01%7Cgcolavizza%40turing.ac.uk%7C101893f2818d458761a408d7abeb720f%7C4395f4a7e4554f958a9f1fbaef6384f9%7C0%7C0%7C637166900034706470&sdata=VSQWB%2BKctF9dEyqcSNltwVHfnJAdUtRZ6qLBIrgdYn4%3D&reserved=0).

Secondly, and to repeat ourselves once more, not reporting (in the abstract, because of course this is in the text and in the code and data) the effect size is done in order to avoid misunderstandings: we use only a fraction of the available citations because we calculate them using PubMed OA data. Hence the effect size is just a fraction of what it would be with total citation counts (approximately one fifth, for PLoS). We test that there is indeed a high correlation between our and the total citation counts, so we conclude that our approach is acceptable. What thus counts is the relative effect we find. We believe doing as the reviewer suggest would be misleading and confusing, and making the readers missing the point. Furthermore, we underline that we take this approach to keep all our results fully reproducible and in order to avoid using proprietary citation data.

There are still lingering issues with the statistics. At this point I’m fairly sure they don’t undermine the main result, but it would good to have them addressed.

First, the mean and median of the h-index are closely correlated (0.82 in Table 5) so the authors are not convincing when they claim they are capturing a different signal (lines 264 to 267). Given the known difficulties in making sense of coefficients for closely correlated variables, they should also refrain from over-interpreting this aspect of the results (“Crucially, the mean author H-index is strongly correlated with higher citations, while not so much the median, indicating the preferential citation advantage given to more popular authors.” Lines 283-284).

This is a minor point that we checked, and can easily be reproduced as well. We could add a further sentence to clarify in the paper.

Second, including an interaction term in the model (das_category*is_plos) means that the coefficients for these variables alone (das_category and is_plos) no longer have a simple interpretation. For example, the coefficient 0.252 for C(das_category)3 in Table 6 only applies to when is_plos is 0 (i.e. just BMC articles). Since the das_category is their main focus, the authors need to address this throughout.

We agree on this and have checked for it already. We can indeed a comment or even add a table and short discussion with a sensitivity analysis if this is preferred.

Third, I still disagree with the authors’ philosophical approach to their analyses and would much prefer that they decide on their statistical model before they collect their data and stick with that. Analysing their data in lots of different ways with many different variables just looks like p-hacking, even if that’s not how the authors perceive it.

We interpret this as the reviewer's own point of view. Furthermore, while we acknowledge prospective study registration can be beneficial (especially in clinical trials), preregistration for this type of study is uncommon and not required by PLoS, or other journals to our knowledge. We do not try to find the best p-value as all our results are significant with all the models and methods we tested thus we do not have a problem with the significance nor the power of our results. This seems to be what the reviewer is also saying: "fairly sure that [the statistics] don't undermine the main result". We would appreciate that claims of p-hacking were substantiated with evidence. Given that all the code and data are shared, the reviewer is free to engage with them to evidence their position but we are not aware they have done this.
